# Quantifying relations and similarities of the meteorological parameters among the weather stations in the Alberta Oil Sands region

**DOI:** 10.1371/journal.pone.0261610

**Published:** 2022-01-13

**Authors:** Dhananjay Deshmukh, M. Razu Ahmed, John Albino Dominic, Mohamed S. Zaghloul, Anil Gupta, Gopal Achari, Quazi K. Hassan

**Affiliations:** 1 Schulich School of Engineering, University of Calgary, Calgary, Alberta, Canada; 2 Resource Stewardship Division, Alberta Environment and Parks, University Research Park, Calgary, Alberta, Canada; Curtin University, AUSTRALIA

## Abstract

Our objective was to quantify the similarity in the meteorological measurements of 17 stations under three weather networks in the Alberta oil sands region. The networks were for climate monitoring under the water quantity program (WQP) and air program, including Meteorological Towers (MT) and Edge Sites (ES). The meteorological parameters were air temperature (AT), relative humidity (RH), solar radiation (SR), barometric pressure (BP), precipitation (PR), and snow depth (SD). Among the various measures implemented for finding correlations in this study, we found that the use of Pearson’s coefficient (r) and absolute average error (AAE) would be sufficient. Also, we applied the percent similarity method upon considering at least 75% of the value in finding the similarity between station pairs. Our results showed that we could optimize the networks by selecting the least number of stations (for each network) to describe the measure-variability in meteorological parameters. We identified that five stations are sufficient for the measurement of AT, one for RH, five for SR, three for BP, seven for PR, and two for SD in the WQP network. For the MT network, six for AT, two for RH, six for SR, and four for PR, and the ES network requires six for AT, three for RH, six for SR, and two for BP. This study could potentially be critical to rationalize/optimize weather networks in the study area.

## 1. Introduction

In general, understanding weather conditions (in other words meteorology) of the atmosphere plays a critical role in our sustainable existence on the Earth surface, including real-time weather analysis for forecasting weather-induced calamities [1,2]. Such analysis is based on the commonly monitored meteorological parameters, including air temperature (AT), relative humidity (RH), solar radiation (SR), atmospheric pressure (or barometric pressure, BP), precipitation (PR; i.e., rain, snow, freezing rain, sleet, hail, drizzle, and fog), and wind (i.e., speed and direction) [3]. These parameters may also be used for other applications. For example, the measurements of AT and BP are used to study the movement of air and energy exchange within the atmosphere. Also, AT and RH are key elements that highly influence the growth of plants and organisms sustaining in a particular region and facilitate the public service and environmental policy [4]. AT is also directly related to the land surface temperature that caused the increasing intensity of surface urban heat islands in many cities [5,6], and help determining temperature regimes in vegetation phenology [7]. On the other hand, SR plays key role in the energy balances of various physical, chemical and biological processes and utilized as most abundant of all renewable energy resources [8]. In addition, the PR measurement has many applications in flood estimation, computing plant water requirements, hydrological analyses and studying water-related issues including erosion and quality [9]. For example, extreme AT and PR events caused warming in the coastal and inland areas [10], and PR records help in forecasting river flow [11]. Moreover, wind speed and direction recordings are often required to issue weather-related warnings, and they also play a major role in the movement and distribution of spores, pollen, and pollution elements in the atmosphere [12,13].

The meteorological parameters are measured by the weather stations in a network to understand the dynamics of weather conditions for a particular region. Weather stations are usually distributed with specific distances to ensure that their measurements and observations are adequate for the broader need of climate-dependent services, applications, and research for a region [14]. These observations require to fulfil certain criteria and accuracies in describing the meteorological parameters. As such, World Meteorological Organization (WHO) provides few guidelines to ensure representativeness of the observations. Such representativeness is often related to space (horizontal spacing between two stations) and time (measurement interval). The horizontal spacing depends several factors including station locations (land or sea), type of recordings (continuous or non-continuous), and spatial scale of the weather prediction model (i.e., global, regional or local) [3]. For example, the horizontal spacing between two stations should not exceed 250 km in a populated area and 300 km in sparsely populated areas for land stations, whereas 250 km for sea stations [15]. In the case of various weather-related models, the guidelines suggest that at least one station is required for each 10000, 2500, and 100 km^2^ area for the Numerical Weather Prediction (NWP) model, Global Model (GM), and Regional Model (RM), respectively [16]. On the other hand, measurement frequency varies depending on the potential applications. For example, minutes interval for aviation, hours for agriculture, and days for climate description (historical record and description of average daily weather events) [3].

Often weather stations are established for specific purposes by different agencies without coordination and following any established guidelines that results in sub-optimal network (with possible overlap) in the region having less or more than the recommended spacing between weather stations [17]. Such overlapping networks in a region, serving different stakeholders, usually involve measurements of the same parameters with near-similar readings, although they are intended for different goals and purposes. However, such overlapped network might result observational redundancy without improving the quality of information, and require higher operating and maintenance cost funded from the same source, i.e., provincial, or federal government [18]. Three distinct networks of weather stations are currently operational in the oil sands region of Alberta under Oil Sands Monitoring (OSM) Program. The program undertakes environmental monitoring within the area that integrates air, water, land, and biodiversity to assess any impacts of oilsands activities on the environment [19]. To establish the relationship between network density and network performance due to such overlapping, research on the rationalization or optimization (i.e., redundancy or gaps in the networks) are found in literature [20,21].

Rationalization of climate monitoring stations have been implementing in Canada over the last three decades, which allowed the reduction of several stations without sacrificing useful details of climatic information [22,23]. However, the approach was often restricted to a maximum of two parameters, such as AT and PR, due to their wider applications in climatic and hydrological modellings [24–27]. Such an approach is determined by capturing and comparing anomalies in the entire network, and its various subsets. These anomalies were estimated using three approaches, such as: (i) time-series trend analysis per decade [20,21]; (ii) statistical analysis of the parameters, such as, mean, median, variance, standard deviation, and coefficient of variation [24,28,29]; and (iii) spatial descriptive statistics in a GIS (Geographic Information System) environment [27,30–32]. In addition to these approaches, closeness and similarity between two datasets are also estimated using two distinct analyses, i.e., graphical, and quantitative [33]. In graphical analysis, the observations of two stations are visually compared for the same period (time-series plot) for identifying time-related variations between two datasets, such as linear or nonlinear trends, upward and downward shifts [34], and presence of error [33]. Additionally, the scatter plot is another graphical representation that is frequently used to test the model performance (closeness between two datasets) by using the coefficient of determination and slope of the fitted line. On the other hand, the quantitative analysis is classified into two categories, such as (i) analysis of association, and (ii) analysis of coincidence.

Analysis of association is the accuracy estimation using indices, such as coefficient of determination, Pearson correlation coefficient, Spearman’s correlation coefficient, Nash-Sutcliffe coefficient, and cosine similarity. Among these indices/measures, Pearson’s correlation coefficient is one of the most widely used statistics today, because it determines both the strength and direction of the relationship between two variables [35]. On the other hand, analysis of coincidence includes several metrices, such as absolute average error (AAE), relative difference (RD), mean squared error (MSE), root mean square error (RMSE), and bias (B). Among these, AAE is a more natural measure of average error in compared to the highly used measure of RMSE. This is because, RMSE is a function of three characteristics of a set of errors, while AAE is relatively simple to calculate [36]. While these methods indicate the closeness and similarity between two datasets and the ability of one dataset to predict another, they do not estimate actual similarity, i.e., the number of similar values (data points) in each station-pair in the datasets. Moreover, to the best of our knowledge from literature, we did not find any approach that considered instrumental errors to quantify data closeness or similarity in finding redundant parameters/stations in the weather networks. Hence, assuming the sufficiently dense weather stations, we set our overall goal to rationalize the existing networks with a new concept of percentage similarity (PS) and identify the number of weather stations and their measured parameters that provides a similar set of observations. For this, performing a parametric similarity analysis for the measured parameters, including AT, RH, SR, BP, PR, and snow depth (SD) would be appropriate. In fulfilling our overall goal, the specific objectives in this study were to:

- evaluate various measures in both the analysis of association and coincidence in finding the most representative measures in establishing the relationship;
- calculate the percentage of similarity of the measurements in the datasets by considering the instrumental errors to find the similarity among the weather stations and meteorological parameters of the networks; and
- determine an optimal network of weather stations in the oil sands region based on the estimation of the percentage of similarity in each meteorological parameter.

## 2. Study area and data availability

### 2.1. Study area

**Fig 1:**
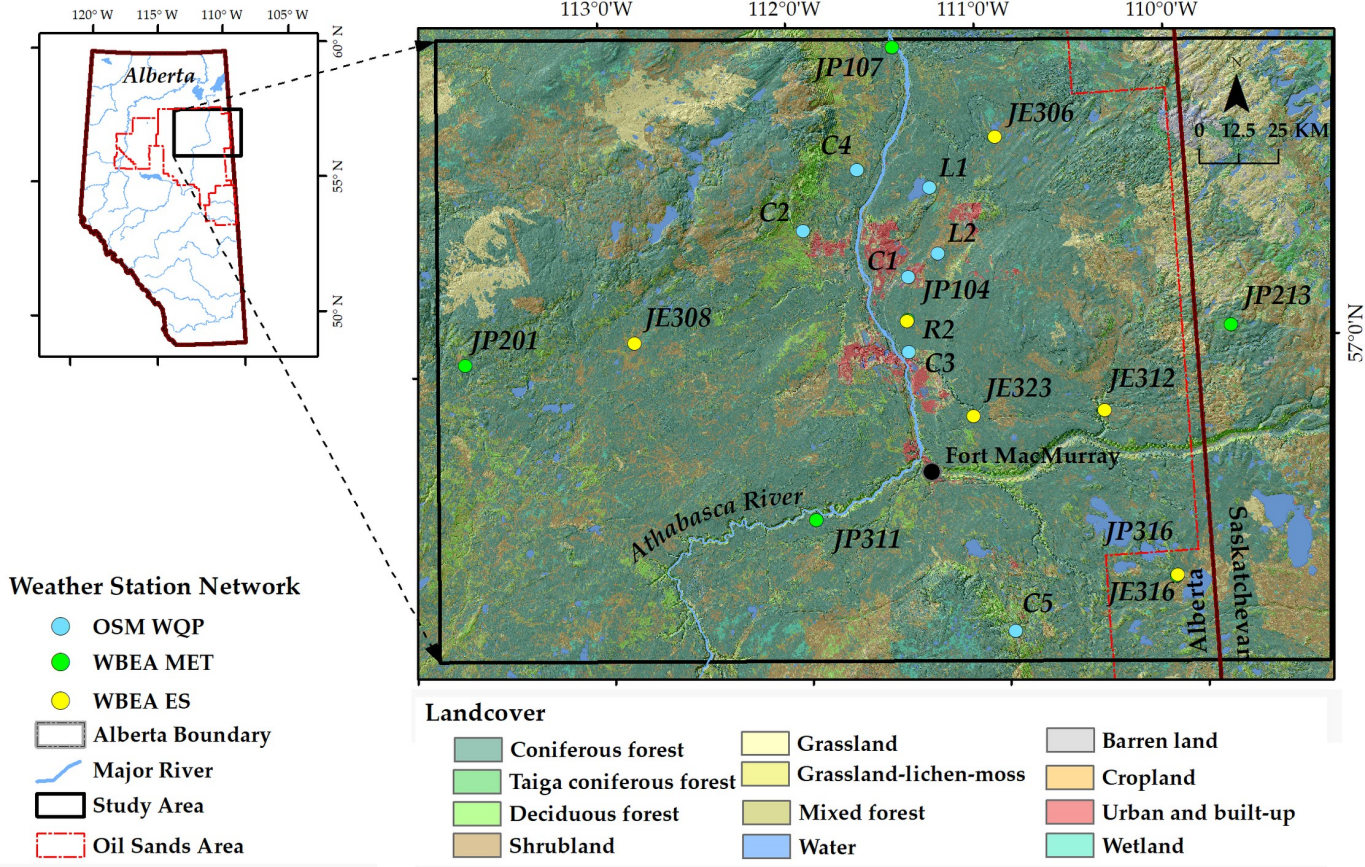
Weather stations of three networks in the study area. A digital elevation model (source: USGS; https://earthexplorer.usgs.gov/; accessed on 15 Nov 2021) was used in the background, and the 2015 landcover classes and the major rivers (source: Government of Canada provided under Open Government Licence–Canada that allows adaptation for any lawful purpose; https://open.canada.ca/data/en/dataset/; accessed on 15 Nov 2021) over it were made semi-transparent to understand the topography.

The lower Athabasca River Basin (ARB) of Northern Alberta in Canada, also a part of the Athabasca oil sands area, is our study area. Three distinct networks of weather stations measure the meteorological parameters in the area (Fig 1). The networks included: (i) OSM Water Quantity Program (WQP): seven stations (i.e., C1, C2, C3, C4, C5, L1 and L2); (ii) WBEA Meteorological Towers (MT): six stations (i.e., JP104, JP107, JP201, JP213, JP311, and JP316); and (iii) WBEA Edge Sites (ES): six stations (i.e., JE306, JE308, JE312, JE316, JE323, and R2 (see Fig 1). These 19 weather stations of the networks span between longitude 109°W and 114°W, and latitude 56°N and 58°N. Note that the minimum and maximum distances between stations in each network were found 11.91 and 153.56 km, 69.31 and 241.83 km, and 36.48 and 186.05 km for OSM WQP, WBEA MT, and WBEA ES, respectively. This sufficiently fulfills the WMO requirement of having networks of stations (i.e., 300 km distance between stations for a sparsely populated land) to represent the study area. The Athabasca River passes through the study area that contains various tributaries of the Athabasca and Clearwater rivers. The landscape varies from upland Boreal forests to poorly drained wetlands within the low land regions [37]. The elevation of the stations in each network varies from 294 to 559 m, 256 to 626 m, and 299 to 520 m msl (mean sea level) for OSM WQP, WBEA MT, and WBEA ES, respectively. The landscape characteristics, i.e., topography, surrounding vegetation and closeness to a water body, of the stations are presented in Table 1.

**Table 1:** Landscape characteristics of the weather stations in the three networks.

Station	Distance (in Km)		River Valley
	Forest	Waterbody	
C1, C4, and JE323	< 0.5 (Coniferous)	> 10	Athabasca
JP316 and JE316		< 10	Not in valley
C3, JP104, JP107, and JP311		< 10	Athabasca
C5, JP201, and JP213	> 0.5 (Coniferous)	> 10	Not in valley
L1, L2, R2, and JE306	< 0.5 (Shrubland)	< 10	Athabasca
C2	< 0.5 (Broadleaf)	> 10	Athabasca
JE308	< 0.5 (Shrubland)	> 10	Not in valley
JE312	< 0.5 (Coniferous)	> 10	Clearwater

The climatic regime of the area is sub-arctic, where the average annual AT varies from 0.7 to 1°C. It is characterized by long and cold winter, short and wet summer, and short spring and fall seasons. Spring and fall are receiving substantial amount of annual total PR that varies from 376 to 456 mm. The wettest month in the region is July, while November through April are the driest months. Based on climate normal from year 1981–2010 at Fort MacMurray, the area is having average annual RH of 40.1 to 87.5%, average BP 96.9 to 97.2 kPa, and average annual SD 0 to 30 cm [38]. Besides, the area receives an average annual SR of 108–128 W/m^2^ [39].

### 2.2. Meteorological parameters and data availability

Stations C1 to C5 of OSM WQP record daily AT in °C, RH in %, SR in W/m^2^, BP in kPa, PR in mm, and SD in cm at 2 m height, where L1 and L2 record daily AT, RH, and PR. On the other hand, WBEA MT stations record hourly AT, RH, SR, and PR at 2 m, where AT and RH are also measured at 16, 21, and 29 m. In case of WBEA ES network, all stations record hourly AT, RH, SR, and BP at 2 m. Wind speed and direction is also measured at these stations, but this variable was not analyzed in the scope of this paper. The available period of data records for the networks are provided in Table 2.

**Table 2:** Data availability of the three networks in the study area, where ‘-’ indicates measurements were not available.

Network	Weather Station	Meteorological Parameter	Period of Records	
			From	To
OSM WQP	C1	AT, SR, and PR RH SD BP	10-March-1988 10-May-1995 26-October-1995 -	31-March-2017 -
	C2	AT, RH, SR, and BP PR SD	16-October-1995 11-June-2009 03-November-2010	31-March-2017
	C3	AT, RH, SR, BP, and SD PR	03-November-2010 13-August-2009	31-March-2017
	C4 and C5	AT, RH, SR, BP, PR, and SD	25-July-2011	31-March-2017
	L1	AT and RH PR	09-February-2007 09-August-2002	31-March-2017
	L2	AT and RH PR	25-September-2007 01-January-2007	31-March-2017
WBEA MT	JP104 and JP201	AT, RH and SR PR	27-May-2014 -	31-January-2019 -
	JP107 and JP213	AT, RH, SR, and PR	29-August-2012	01-April-2018
	JP311 and JP316		30-July-2013	
WBEA ES	JE306, JE308 and JE312	AT, RH, SR, and BP	25-March-2014	01-April-2019
	JE316 and JE323		07-March-2014	
	R2		24-January-2011	

## 3. Methods

### 3.1. Analysis of association

We applied several measures including Pearson correlation coefficient (*r*), coefficient of determination (*R*^*2*^), Spearman’s correlation coefficient (*R_s_*), Nash-Sutcliffe efficiency (*E*), and Cosine similarity (*Cosθ*), as shown in Eqs 1 to 5.

$$r=\left[\frac{\sum_{i=1}^{n}\left({D1}_{i}-\bar{D1})({D2}_{i}-\bar{D2}\right)}{\sqrt{\sum_{i=1}^{n}{\left({D1}_{i}-\bar{D1}\right)}^{2}}\sqrt{\sum_{i=1}^{n}{\left({D2}_{i}-\bar{D2}\right)}^{2}}}\right]$$ (1)

$$R^{2}=1-\frac{RSS}{TSS}$$ (2)

$$R_{s}=1‐\left[\frac{6\sum_{i=1}^{n}D^{2}}{n^{3}-n}\right]$$ (3)

$$E=1-\left[\frac{\sum_{i=1}^{n}{\left(D1-D2\right)}^{2}}{\sum_{i=1}^{n}{\left(D1-\bar{D1}\right)}^{2}}\right]$$ (4)

$$Cos\theta =\frac{\sum_{i=1}^{n}D1D2}{\sqrt{\sum_{i=1}^{n}{D1}^{2}}\sqrt{\sum_{i=1}^{n}{D2}^{2}}}$$ (5)

where D1, and D2 refers to observational data recorded at Station A and B, respectively, n is the number of observations, *RSS* is the residual sum of squares, and *TSS* is the total sum of squares.

### 3.2. Analysis of coincidence

We adopted several measures including AAE, RD, MSE, RMSE, and B, as shown in Eqs 6 to 10. Note that the meaning of all symbols in these equations are same as described in sub-section 3.1. Analysis of Association.


$$AAE=\frac{1}{n}\sum_{i=1}^{n}\left|\left({D1}_{i}-{D2}_{i}\right)\right|$$ (6)

$$\mathrm{RD}=\frac{100}{n}\sum_{i=1}^{n}\frac{\left|{D1}_{i}-{D2}_{i}\right|}{\max\left(|{D1}_{i}|,|{D2}_{i}|\right)}$$ (7)

$$\mathrm{MSE}=\frac{1}{n}\sum_{i=1}^{n}{\left({D1}_{i}-{D2}_{i}\right)}^{2}$$ (8)

$$\mathrm{RMSE}=\sqrt{\frac{1}{n}\sum_{i=1}^{n}{\left({D1}_{i}-{D2}_{i}\right)}^{2}}$$ (9)

$$B=\frac{1}{n}\sum_{i=1}^{n}\left({D1}_{i}-{D2}_{i}\right)$$ (10)

### 3.3. Determination of the representative measure

It might be possible that one estimate from each of the association and coincidence measures could sufficiently describe the similarities in meteorological observations to represent the entire datasets. Identifying one such measure would reduce the ambiguity in using multiple measures. Therefore, to find such a representative measure in each group, we performed linear regressions among measures. In case of the association, where all measures were dimensionless, we performed the comparison of the estimates from all meteorological parameters together. In contrast for the measures of coincidence, we separately plotted the estimates derived for various meteorological parameters as the estimates were in different units.

### 3.4. Similarity analysis

We performed a similarity analysis on the station pairs for the variables (i.e., meteorological parameters) using acceptable values of the instrumental error suggested by the standard operating procedure (SOP; [40–43]) by applying Eq 11.

$$\mathrm{PS}=\frac{N2}{N1}\times 100$$ (11)

where N1 is the total data count, and N2 is the count that satisfy the following set of arguments:

- If the absolute difference between D1 and D2 are ± 0.5°C for AT, ± 5% for RH, and ± 2.5 cm for SD as suggested in the SOP; and
- If the % deviation between D1 and D2 falls 20% for hourly SR, 10% for daily SR, 2% for PR, and 1% for BP as per the SOP. In this case, the deviation is calculated based on the higher value between D1 and D2.

## 4. Results and discussion

### 4.1. Comparison among the measures of association

**Fig 2:**
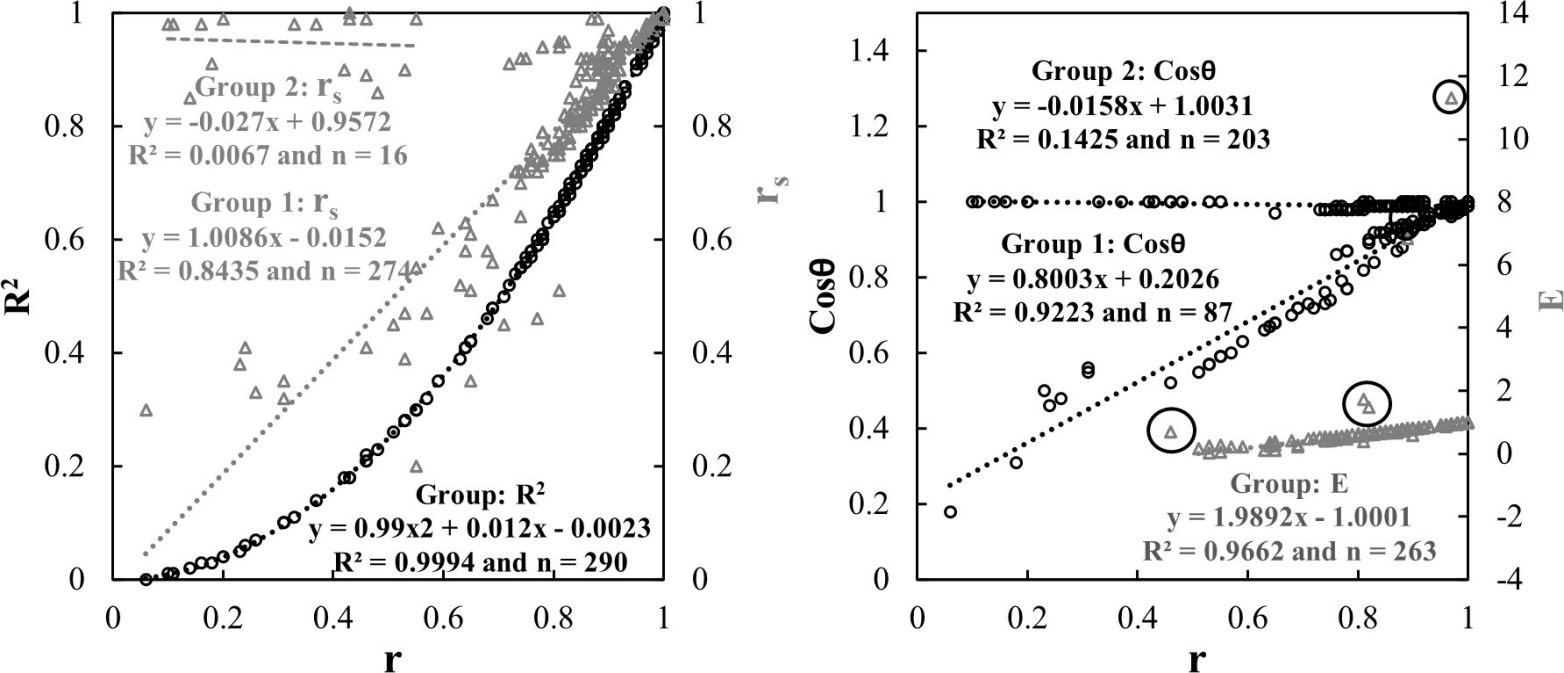
Measures of association related relationships among **(a)** *r*, *R*^*2*^ and *R_s_*; and **(b)** *r*, *Cosθ* and *E* for the entire dataset. Data outliers are Group 2 in both panels and circled in panel **(b)**.

Fig 2 shows the plots of association measures that were estimated for all the meteorological parameters with reference to *r*. Our analyses revealed that the association between two datasets could be described by using only *r*, as high correlations (*R*^*2*^ > 0.84) were observed between r and other measures (i.e., *R*^*2*^, *R*_*s*_, *Cosθ*, *E*) in most cases. The *R*^*2*^ indicates the proportion of variance in one variable due to another. In general, values of *R*^*2*^ if higher than 0.50 were considered as significant and acceptable [44,45] and values of *R*^*2*^ higher than 0.70 were considered as strong [46,47]. In case of *E*, we considered only the positive estimates as negative estimates would indicate that the observed mean would be a better predictor in this context [48]. Consequently, we opted to use *r* as a representative for estimating the measures of association for the entire datasets.

### 4.2. Comparison among measures of coincidence

**Fig 3:**
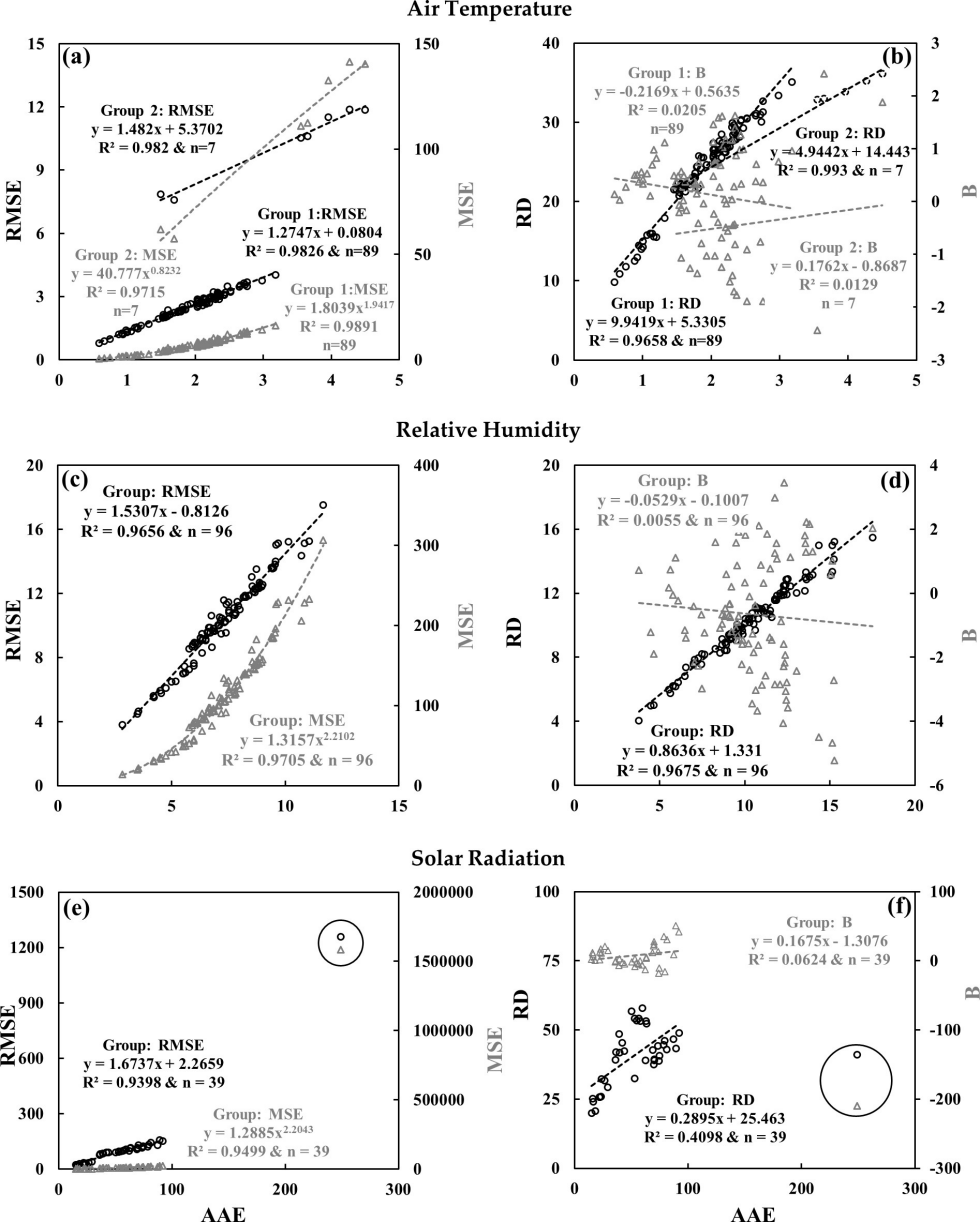
Measures of coincidence related relationships among **(a)** AAE, RMSE and MSE, and **(b)** AAE, RD and B for AT in °C; **(c)** AAE, RMSE and MSE, and **(d)** AAE, RD and B for RH in %; **(e)** AAE, RMSE and MSE, and **(f)** AAE, RD and B for SR in W/m^2^ for the entire dataset. Data outliers are circled in panels **(e)** and **(f)**.

**Fig 4:**
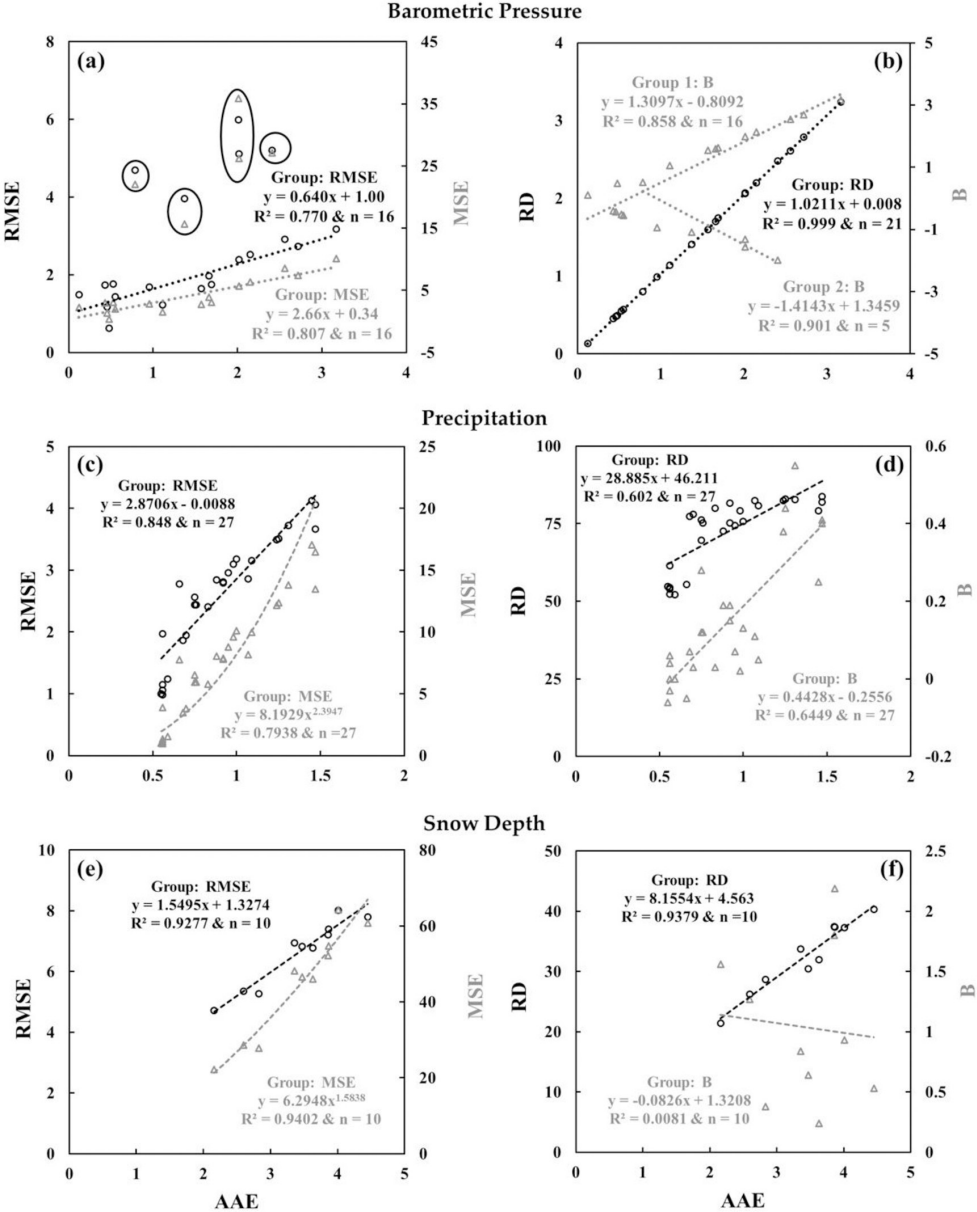
Measures of coincidence related relationships among **(a)** AAE, RMSE and MSE, and **(b)** AAE, RD and B for BP in kPa; **(c)** AAE, RMSE and MSE, and **(d)** AAE, RD and B for PR in mm; and **(e)** AAE, RMSE and MSE, and **(f)** AAE, RD and B for SD in cm for the entire dataset. Data outliers are circled in panel **(a)**.

For the comparison, we plotted AAE against other relevant measures (i.e., RMSE, MSE, RD and B) for various meteorological parameters (see Figs 3 and 4). In general, the AAE estimates were well correlated with strong *R*^*2*^ values (> 0.96) with all other estimates except for RD and B. It would be the case as RD provided an estimate of percent deviation from the highest record between the two stations for a parameter of interest, while AAE, RMSE and MSE provided the actual differences. On the other hand, B presented the summation of both negative and positive differences between two data records; thus it would potentially give different values in comparison to AAE, RMSE and MSE [49]. Taking these into consideration, we assumed to employ AAE as a representative for estimating the measures of coincidence for the entire datasets.

### 4.3. Relations and similarity analysis

We determined the relations and similarity for each station-pair for the associated parameters according to the estimated values of *r*, AAE, and PS. Here, we considered at least 75% for PS in finding the similarity between station-pairs. Estimated values of *r*, AAE, and PS for each station-pair of the three networks are shown in Tables 3–6, and associated regression equations are shown in Supporting Information. A detailed relations and similarity analysis for identifying the least number of required station/s in each network for each meteorological parameter are presented in the following sub-sections. Note that the analysis was performed in each network separately (instead of all three networks combined) and we considered it as a limitation in this study. It was because, we wanted to retain the different goals and purposes for establishing three distinct networks by the agencies.

**Table 3:** Relation and similarity analysis of all meteorological parameters of interest for OSM WQP stations.

Station Pair		AT				RH				SR				BP				PR				SD			
		n	*r*	AAE (°C)	PS (%)	n	*r*	AAE (%)	PS (%)	n	*r*	AAE (W/m^2^)	PS (%)	n	*r*	AAE (kPa)	PS (%)	n	*r*	AAE (mm)	PS (%)	n	*r*	AAE (cm)	PS (%)
C1 vs	C2	2943	1.00	0.94	64.39	2943	0.91	4.99	**86.95**	2869	0.96	26.52	25.04	-	-	-	-	2726	0.59	0.95	5.81	2611	0.93	3.47	**77.48**
	C3	2338	1.00	0.59	**84.60**	2338	0.97	2.83	**98.08**	2338	0.98	15.99	36.17	-	-	-	-	2626	0.83	0.56	9.79	2327	0.96	2.60	**81.52**
	C4	2076	1.00	1.16	53.76	2076	0.88	5.76	**83.00**	2076	0.97	16.41	32.38	-	-	-	-	2076	0.69	0.88	5.69	2076	0.95	2.83	**76.40**
	C5	1993	0.99	1.79	36.43	1993	0.83	6.77	**77.57**	1993	0.95	23.68	22.83	-	-	-	-	1993	0.63	1.25	4.42	1993	0.92	3.87	70.10
	L1	3394	1.00	0.92	68.56	3340	0.91	5.18	**88.17**	-	-	-	-	-	-	-	-	3974	0.68	0.76	4.67	-	-	-	-
	L2	3406	1.00	0.66	**81.03**	3298	0.96	3.49	**97.67**	-	-	-	-	-	-	-	-	3131	0.81	0.70	4.73	-	-	-	-
C2 vs	C3	2322	1.00	1.06	58.05	2322	0.90	5.49	**84.07**	2322	0.96	22.32	33.96	2322	0.81	1.11	17.74	2616	0.55	0.98	3.01	2037	0.93	3.63	72.26
	C4	2060	1.00	0.98	67.28	2060	0.92	4.55	**91.41**	2060	0.96	23.10	29.84	2060	0.82	1.57	0.44	2002	0.69	0.92	8.85	1786	0.97	2.16	**85.95**
	C5	1993	0.99	1.56	45.51	1993	0.84	6.34	**80.13**	1993	0.92	29.35	23.95	1993	0.81	1.69	1.46	1935	0.46	1.47	6.64	1719	0.89	4.45	70.27
	L1	2823	0.99	1.11	59.69	2841	0.89	5.53	**85.46**	-	-	-	-	-	-	-	-	2588	0.57	0.92	2.91	-	-	-	-
	L2	3002	1.00	1.00	62.09	2932	0.92	4.74	**90.79**	-	-	-	-	-	-	-	-	2658	0.53	1.09	5.07	-	-	-	-
C3 vs	C4	2076	1.00	1.32	49.37	2076	0.88	5.97	**81.60**	2076	0.97	15.09	42.29	2076	0.90	0.48	**98.41**	2020	0.64	1.00	4.53	2065	0.89	4.01	72.59
	C5	1993	0.99	1.59	40.99	1993	0.86	5.98	**81.44**	1993	0.95	18.30	36.93	1993	0.89	2.72	0.30	1993	0.65	1.24	5.05	1982	0.92	3.36	**75.58**
	L1	2322	1.00	1.14	59.52	2322	0.89	5.61	**84.15**	-	-	-	-	-	-	-	-	2548	0.64	0.75	5.03	-	-	-	-
	L2	2338	1.00	0.75	**76.22**	2337	0.96	3.53	**96.15**	-	-	-	-	-	-	-	-	2556	0.71	0.83	3.99	-	-	-	-
C4 vs	C5	1993	0.99	2.03	37.03	1993	0.77	7.18	73.81	1993	0.93	22.16	30.71	1993	0.97	3.17	0.00	1993	0.51	1.45	9.69	1993	0.91	3.86	72.70
	L1	2060	1.00	1.00	62.82	2060	0.91	4.21	**92.14**	-	-	-	-	-	-	-	-	1988	0.74	0.75	8.45	-	-	-	-
	L2	2076	1.00	1.20	57.18	2075	0.91	4.50	**90.17**	-	-	-	-	-	-	-	-	1958	0.65	1.07	4.51	-	-	-	-
C5 vs	L1	1977	0.99	2.09	33.99	1977	0.76	7.38	71.52	-	-	-	-	-	-	-	-	1905	0.53	1.31	7.23	-	-	-	-
	L2	1993	0.99	1.75	40.69	1992	0.86	5.56	**83.99**	-	-	-	-	-	-	-	-	1875	0.55	1.47	6.98	-	-	-	-
L1 vs	L2	3227	1.00	0.88	68.79	3116	0.92	4.23	**92.49**	-	-	-	-	-	-	-	-	3083	0.77	0.68	9.25	-	-	-	-

Here, ‘-’ indicates that measurements were not available, and bold highlighted values indicate strong PS (i.e., ≥ 75%).

**Table 4:** Relation and similarity analysis of AT and RH at different heights for WBEA MT stations.

AT																	
Station Pair		2 m				16 m				21 m				29 m			
		n	*r*	AAE	PS	n	*r*	AAE	PS	n	*r*	AAE	PS	n	*r*	AAE	PS
JP104 vs	JP107	32402	0.98	2.05	34.48	32110	0.99	1.77	37.66	32217	0.99	1.69	39.79	32456	0.99	1.62	41.52
	JP201	39971	0.78	3.65	23.50	39915	0.98	2.43	24.35	37881	0.98	2.30	25.00	39886	0.99	2.05	28.55
	JP213	30436	0.98	2.35	26.16	32812	0.99	2.16	27.02	30015	0.99	2.03	29.07	32284	0.99	2.11	27.18
	JP311	32675	0.98	1.89	36.11	32701	0.99	1.64	40.52	32198	0.99	1.56	41.90	31637	0.99	1.53	42.81
	JP316	32153	0.98	2.14	30.06	31914	0.99	1.85	34.54	31554	0.99	1.77	36.19	32065	0.99	1.74	36.54
JP107 vs	JP201	32622	0.74	4.5	20.21	32570	0.97	3.18	20.30	31787	0.97	2.98	21.39	32946	0.97	2.76	22.72
	JP213	43687	0.98	2.42	25.59	43360	0.99	2.35	24.07	41529	0.99	2.17	26.40	42704	0.99	2.21	25.15
	JP311	37515	0.97	2.72	26.75	36898	0.98	2.32	29.35	37476	0.98	2.24	30.70	35534	0.98	2.21	30.89
	JP316	42816	0.97	2.73	24.66	39599	0.98	2.30	28.91	33844	0.98	2.22	30.29	35280	0.98	2.20	30.23
JP201 vs	JP213	30758	0.72	4.27	24.18	33282	0.97	2.65	26.47	29601	0.97	2.44	28.35	32783	0.98	2.36	29.47
	JP311	33076	0.78	3.55	26.84	33168	0.98	2.31	28.24	31700	0.98	2.15	29.36	32103	0.99	1.86	33.73
	JP316	32379	0.75	3.96	24.81	32204	0.97	2.52	26.93	30947	0.98	2.35	28.26	32384	0.98	2.10	31.11
JP213 vs	JP311	37125	0.98	2.76	25.36	39799	0.98	2.52	25.75	37612	0.99	2.33	27.88	38334	0.99	2.37	27.21
	JP316	40756	0.99	1.93	37.51	40869	0.99	1.80	36.26	33858	0.99	1.56	41.42	36705	0.88	1.69	38.81
JP311 vs	JP316	36401	0.99	1.82	36.54	35670	0.99	1.52	43.47	33733	0.99	1.46	45.16	33610	0.87	1.49	46.52
**RH**																	
JP104 vs	JP107	32413	0.87	7.09	**76.77**	32109	0.88	7.05	**76.36**	30123	0.88	6.70	**78.33**	32454	0.84	7.41	**76.19**
	JP201	39971	0.81	8.53	71.54	39915	0.85	8.27	70.51	37881	0.84	8.69	69.02	39886	0.83	8.54	70.06
	JP213	32610	0.88	6.63	**78.71**	32814	0.88	6.69	**78.06**	30015	0.88	6.85	**77.50**	31828	0.85	7.49	**75.81**
	JP311	32031	0.89	6.61	**78.75**	32701	0.90	6.27	**79.96**	31468	0.90	6.36	**80.06**	31536	0.86	6.75	**79.30**
	JP316	32154	0.86	7.11	**76.86**	31897	0.87	7.20	**76.30**	31551	0.87	7.15	**76.48**	29903	0.84	7.30	**76.40**
JP107 vs	JP201	32633	0.74	10.14	65.04	32569	0.74	10.85	59.59	29695	0.75	11.06	58.05	32944	0.76	10.72	58.32
	JP213	46089	0.84	7.52	74.91	43356	0.86	7.81	71.95	38330	0.86	7.51	73.52	42240	0.85	7.78	72.12
	JP311	36882	0.81	8.60	69.63	36893	0.81	8.79	68.31	34632	0.81	8.74	69.34	35431	0.80	8.84	68.44
	JP316	42897	0.80	8.68	69.30	39538	0.81	8.82	68.06	31749	0.82	8.95	67.72	33110	0.81	9.00	67.12
JP201 vs	JP213	33064	0.74	9.68	68.34	33284	0.77	9.57	66.36	29601	0.78	9.39	67.10	32327	0.78	9.47	66.71
	JP311	32474	0.79	8.73	71.09	33168	0.84	8.23	70.27	30959	0.83	8.66	69.08	32002	0.86	7.79	72.47
	JP316	32380	0.73	9.60	68.78	32187	0.78	9.43	66.67	30944	0.78	9.56	66.50	30225	0.80	8.87	68.65
JP213 vs	JP311	38884	0.83	7.89	72.66	39804	0.82	8.22	71.09	34086	0.82	8.16	71.99	37777	0.82	8.39	70.16
	JP316	43515	0.90	5.92	**82.08**	40821	0.90	6.06	**81.42**	33778	0.90	6.13	**81.60**	34073	0.90	6.36	**80.07**
JP311 vs	JP316	36141	0.88	6.35	**79.99**	35622	0.89	6.56	**79.44**	32989	0.89	6.58	**79.29**	31335	0.89	6.28	**80.46**

Here AAE is in °C and % for AT and RH respectively, and PS is in %. Here, bold highlighted values indicate strong PS (i.e., ≥ 75%).

**Table 5:** Relation and similarity analysis of SR and PR for WBEA MT stations.

Station pair		SR				PR			
		n	*r*	AAE (W/m^2^)	PS (%)	n	*r*	AAE (mm)	PS (%)
JP104 vs	JP107	28677	0.90	50.39	26.67	-	-	-	-
	JP201	29879	0.18	249.04	37.67	-	-	-	-
	JP213	25871	0.89	54.75	28.13	-	-	-	-
	JP311	27329	0.90	53.02	27.53	-	-	-	-
	JP316	25681	0.88	56.51	26.95	-	-	-	-
JP107 vs	JP201	27633	0.86	59.58	25.28	-	-	-	-
	JP213	40963	0.91	36.44	42.19	45757	0.26	0.56	2.40
	JP311	34763	0.90	39.13	40.72	38185	0.31	0.56	2.73
	JP316	38882	0.90	42.21	37.44	42837	0.06	0.66	2.35
JP201 vs	JP213	25402	0.85	63.39	27.30	-	-	-	-
	JP311	26604	0.88	57.80	27.51	-	-	-	-
	JP316	25262	0.86	63.04	26.85	-	-	-	-
JP213 vs	JP311	32881	0.90	43.98	38.62	38769	0.31	0.55	2.30
	JP316	37505	0.93	35.98	45.83	42704	0.24	0.59	2.45
JP311 vs	JP316	31889	0.92	39.51	42.74	36939	0.23	0.56	-

Here, ‘-’ indicates that measurements were not available; and bold highlighted values indicate strong PS (i.e., ≥ 75%).

**Table 6:** Relation and similarity analysis of AT, RH, SR, and BP for WBEA ES stations.

Station Pair		AT				RH				SR				BP			
		n	*r*	AAE (°C)	PS (%)	n	*r*	AAE (%)	PS (%)	n	*r*	AAE (W/m^2^)	PS (%)	n	*r*	AAE (kPa)	PS (%)
JE306 vs	JE308	40875	0.97	2.66	26.41	40875	0.82	8.66	68.43	40922	0.85	74.34	35.20	40902	0.42	2.56	3.97
	JE312	41838	0.98	2.03	34.20	28813	0.88	7.31	**75.18**	42063	0.89	53.04	42.54	26124	0.48	2.02	6.46
	JE316	41407	0.98	2.33	31.57	40392	0.84	7.93	71.70	41560	0.84	74.67	34.37	40640	0.46	2.15	4.16
	JE323	40852	0.97	2.47	32.32	40849	0.86	7.99	73.29	41056	0.78	81.32	33.80	41034	0.53	1.66	4.11
	R2	23847	0.99	1.81	40.50	22499	0.89	6.49	**80.60**	31686	0.86	73.52	31.57	23860	0.14	0.79	**92.85**
JE308 vs	JE312	40860	0.98	2.04	35.68	28762	0.86	7.53	74.61	40978	0.87	69.65	36.93	25053	0.37	0.53	**99.94**
	JE316	40427	0.98	2.34	31.08	39414	0.84	7.74	72.99	40473	0.83	87.15	28.53	39563	0.33	0.43	**99.97**
	JE323	39806	0.98	2.12	35.43	39806	0.87	7.36	74.72	39852	0.78	89.23	34.11	39841	0.43	0.95	56.99
	R2	23803	0.98	2.24	30.66	22455	0.89	6.89	**76.90**	31679	0.88	78.61	30.95	23858	0.10	2.41	0.00
JE312 vs	JE316	41546	0.99	1.53	47.83	27354	0.91	5.90	**81.43**	41618	0.90	62.34	36.78	24790	0.43	0.12	**99.99**
	JE323	40925	0.99	1.60	49.72	27749	0.92	5.77	**84.26**	40997	0.82	70.22	39.54	25064	0.55	0.46	**100**
	R2	23905	0.99	2.05	32.01	16863	0.65	11.67	61.42	31744	0.88	68.86	34.12	14604	0.16	2.01	0.00
JE316 vs	JE323	41885	0.98	2.03	39.55	40915	0.89	6.49	**79.83**	41885	0.76	92.06	30.64	40750	0.46	0.55	**99.99**
	R2	23837	0.98	2.15	31.23	21615	0.88	6.82	**78.14**	31674	0.87	70.08	34.48	22517	0.11	2.02	0.00
JE323 vs	R2	23495	0.98	2.27	35.78	21915	0.91	5.99	**81.67**	31338	0.82	79.42	32.46	23496	0.20	1.37	0.18

Here, bold highlighted values indicate strong PS (i.e., ≥ 75%).

#### 4.3.1. OSM WQP network

Table 3 shows relations and similarity analysis on 21 station-pairs for the six associated meteorological parameters (i.e., AT, RH, SR, BP, PR, and SD) in this network. For AT, we found that all the station-pairs were strongly related (i.e., *r* ≥ 0.99 and AAE between 0.59 and 2.09°C), while the PS values were in the range of 33.99 to 84.60%. Considering the PS values, we found that at least five stations (i.e., C1, C2, C4, C5, and L1) would be required for representing this network. The other two stations (i.e., C3 and L2) were similar to C1 station, which would be likely due to their spatial closeness (see Fig 1) and similar altitude (303 to 331 m msl) [50].

For RH, we observed that all the station-pairs were highly related with a *r* ≥ 0.83 and AAE values within the acceptable range of SOP (i.e., less than 10% [41]; see Table 3). We also noticed that the PS values in the station-pairs were greater than 80% except the C4 vs C5 and C5 vs L1. Considering the PS values, we identified that only one station (i.e., C1 with the longest data records) would be required for representing this network. Such higher similarity was observed possibly because they were very close to waterbodies [51]. Note that station L2 was giving the highest PS value, however, this station did not record all meteorological parameters.

In case of SR, we observed that all the station-pairs were very strongly related with a *r* values ≥ 0.92 and low AAE values ≤ 29.35 W/m^2^ (see Table 3). However, the PS values were less than or equal to 42.29%, and therefore, we considered that there was no acceptable similarity in related to SR among the stations, and at least five stations (i.e., C1, C2, C3, C4, and C5) would be required in the network. Note that two stations (i.e., L1 and L2) did not record SR measurements. Such dissimilarity would probably be attributed to several factors, including altitude, terrain, air quality, cloud cover, and vegetation that affect the amount of SR received at any place [52].

In case of the parameter BP, we found that four stations (i.e., C2, C3, C4, and C5) were recording the data. These station-pairs showed their strong relationships with a *r* values ≥ 0.81 and AAE values in the range of 0.48 to 3.17 kPa (see Table 3). Considering the PS values, we identified that at least three stations (i.e., C2, C3, and C5) would be required for representing this network (see Table 3). The other station (i.e., C4) were similar to C3 station (i.e., PS = 98.41%), which would be likely due to their similar altitudes (i.e., 295 and 305 m msl) [53].

Further, in case of PR, we noticed that the station-pairs were having reasonable relations as reflected in *r* (between 0.46 and 0.83) and AAE values (between 0.56 and 1.47 mm) (see Table 3). However, PS values were extremely low for all station-pairs (i.e., between 2.91 and 9.79%); where such a high dissimilarity in the network was due to the variable nature of PR that was evident even at any small scale [9]. This also suggested that all seven stations (i.e., C1, C2, C3, C4, C5, L1, and L2) would be required for representing PR in this network.

Finally in case of SD, station C1 showed very strong relations (i.e., ≥ 0.92) and least error (i.e., AAE ≤ 3.87 cm) among the four station-pairs (see Table 3). Besides, we found that PS values of the three station-pairs with C1 were strong (i.e., > 76%; see Table 3), except C5 station with PS value of 70.10%. These measures indicated that C1 would be a representative for SD in the network for three stations, i.e., C2, C3, and C4. Here, such strong similarity would be due to having little altitude differences in locations of the stations, which attributed to the fact that snow accumulation on the ground was depended on latitude and time of year in addition to altitude, vegetation and wind [54]. Moreover, dissimilarity of C5 (altitude 559 m msl) with C1 station (altitude 303 m msl) would be due to the same fact of having higher elevation differences of the locations. Therefore, both C1 and C5 stations would be the least required ones for SD in the network.

#### 4.3.2. WBEA MT network

Tables 4 and 5 show the relations and similarity analysis of six stations (i.e., JP104, JP107, JP201, JP213, JP311, and JP316) for AT, RH, SR, and PR parameters, where AT and RH were measured at different heights (i.e., 2, 16, 21, and 29 m; see Table 4). In this network, we found that all station-pairs were highly related with *r* values from 0.72 to 0.99 and AAE values ranges from 1.46 to 4.5°C (see Table 4). However, the PS values were significantly low (i.e., between 20.30 to 46.52%) for all station-pairs. Considering the PS values, all stations were required in the network for AT measurements. Such low similarity in the network would be due to the largely spaced distribution of the stations (see Fig 1), and significant altitude differences among these stations (i.e., 256 to 626 m msl) [50].

In the view for RH, all station-pairs showed reasonably strong relations with *r* values from 0.73 to 0.90 and AAE values in the range of 5.92 to 11.06% (see Table 4). Considering the PS values, we identified that at least two stations (i.e., JP104 and JP201) would be required for representing the RH this network. The other stations (i.e., JP107, JP213, JP311, and JP316) were similar to JP104 station (i.e., PS values in the range of 75.81 to 82.08%). Note that strong similarity for RH in this network was observed, because RH value would be similar over a region of interest [55].

Next, in case of SR, we found that all station-pairs in the network were strongly related with the *r* values in the range of 0.85 to 0.93 and low AAE values ranges from 35.98 to 63.39 W/m^2^, except the station-pair JP104 vs JP201 with *r* = 0.18 and AAE = 249.04 W/m^2^ (see Table 5). However, we noticed that PS values were significantly low (i.e., ≤ 45.83%) for all station-pairs, and therefore, all stations (i.e., JP104, JP107, JP201, JP213, JP311, and JP316) were required for the measurements of SR in the network. Such significant dissimilarity in SR was attributed to the factors including varying topography (i.e., high differences in the station-altitudes from 256 m to 626 m msl), and cloud cover that might affected the amount of SR received [52].

Lastly for PR, we found that four stations (i.e., JP107, JP213, JP311, and JP316) were recording the data. Here, all stations-pairs were having weak relations with *r* values ≤ 0.31 and AAE values in the range of 0.55 to 0.66 mm (see Table 5). In addition, we observed that the PS values were extremely low (i.e., ≤ 2.73%), and therefore, all stations were required for the measurements of PR in the network. Such dissimilarity was likely due to the variable nature of PR observed even in a short distances [9].

#### 4.3.3. WBEA ES network

We presented relations and similarity analysis for the six stations (i.e., JE306, JE308, JE312, JE316, JE323, and R2) of WBEA ES network in Table 6. For AT measurements, we found that all station-pairs were having very strong relations with *r* values ranges from 0.97 to 0.99 and acceptable AAE values in the range of 1.53 to 2.66°C (see Table 6) [56]. However, the PS values were low, i.e., in the range of 26.41 to 49.72%. Considering the PS values, all six stations were required in the network. Here, such a dissimilarity was observed probably due to the widely spaced distribution of the stations (see Fig 1), and significant altitude differences [50] that ranged from 299 m to 520 m msl.

In case of RH, we identified that all station-pairs were having very strong relations with the values of *r* ranges from 0.82 to 0.92 and AAE in the range of 5.77 to 8.66% except one station-pair (JE312 vs R2) having a reasonable relation (i.e., *r* = 0.65 and AAE = 11.67%; see Table 6). Here, the PS values were in the range of 61.42 to 84.26%. Considering the PS values, we identified that JE306, JE308 and JE323 stations were required for the measurements of RH in the network. Overall, the similarity of the stations in this network was likely related to having similar landcover [55].

While comparing station-pairs for the measurements of SR, we observed that all were showing strong relations with the *r* values ranges from 0.76 to 0.90 and AAE values in the range of 53.04 to 92.06 W/m^2^ (see Table 6). However, in all cases, we noticed that PS values were considerably low (i.e., ≤ 42.54%). Considering the PS values, we identified that all stations were necessary for the measurements of SR in the network. Such a low similarity would be due to the several factors, including varying topography and cloud cover that could affect receiving amount of SR over a place [52].

In the event of BP, we noticed that the relations were weak to moderate (i.e., *r* values range from 0.11 to 0.55 and AAE values in the range of 0.12 to 2.56 kPa) for all station-pairs in the network (see Table 6). Here, the PS values covered a wide range, i.e., 0 to 99.99%. Considering the PS values, we found that two stations (i.e., JE306 and JE312) were required for measuring BP in the network. Here, strong similarities were likely due to their locations in the valley region with similar altitudes [50].

### 4.4. Suggested optimization

We synthesized the network-specific required weather stations for each of the meteorological parameters (see Table 7) based on similarity analysis detailed in Section 3.4 and 4.3. We observed that all the stations were required for some of the parameters, i.e., (i) PR for OSM WQP, (ii) AT, SR, and PR for WBEA MT; and (iii) AT, and SR for WBEA ES networks. Though, each of the networks might be optimized for some of the parameters but not for all; thus, we would require keeping all the existing stations. As these would be cases, then we would consider not to remove the parameter-specific sensors that even showed redundancy. It would be due to their useability in case of failure of similar sensors in other stations.

**Table 7:** Network-specific required stations for each of the meteorological parameters.

Network	Station ID	Meteorological parameter					
		AT	RH	SR	BP	PR	SD
OSM WQP	C1						
	C2		C1				C1
	C3	C1	C1				C1
	C4		C1		C3		C1
	C5		C1				
	L1		C1				
	L2	C1	C1				
WBEA MT	JP104						
	JP107		JP104				
	JP201						
	JP213		JP104				
	JP311		JP104				
	JP316		JP104				
WBEA ES	JE306						
	JE308				JE312		
	JE312		JE323				
	JE316		JE323		JE312		
	JE323				JE312		
	R2		JE323		JE306		

Color Legend

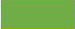
 Station is required to capture spatial variability in meteorological parameter

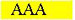
 Meteorological parameter shows at 70% similarity with station ‘AAA’

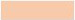
 There is no sensor for recording the meteorological parameter of interest

## 5. Conclusions

In this study, we demonstrated that the PS analysis could quantify similarity between weather stations. It determines the least number of station/s to fully represent the spatial variability in climate measurements required in a network of interest. Moreover, such similarity analysis is a better measure in compared to the relational measures like *r* and AAE in quantifying the similarity between two meteorological datasets. It also assists to identify the best representative station for a particular parameter in a network that could represent the area. In most of the instances, we noticed that the station-pairs were related. However, in case of similarity, we identified that the measurements from five, three, two, and one station/s would be the least required for the measurements of AT, BP, SD, and RH parameters, respectively in the OSM WQP network. For the same network, we also found that seven and five stations were the least required ones for the measurements of SR and PR, respectively. For the WBEA MT network, we identified that all six stations were required for the measurements of AT, SR, and PR, where two would be sufficient for RH. Moreover, in case of WBEA ES network, we found that the measurements from all stations were the least required ones for AT and SR parameters. However, only three stations would be adequate for RH, and two for BP, in this network. Note that we could not perform similarity analysis on few station-pairs, because some stations did not record some specific meteorological parameters of our interest. Nevertheless, we showed that the similarity analysis of using the PS value had potential applications to rationalize/optimize weather station network (stations and parameters) in the study area. It would help to minimize the associated operational costs without sacrificing the scientific credibility of the monitoring programs. However, we recommend evaluating these methods thoroughly before applying them to other weather networks in Canada, and elsewhere during any decision-making process. Further, apart from meteorological study, PS could be a tool to find similarity between two datasets with same parameter in other field of research.

## Supporting information

S1 Table Regression equations in relation to similarity analysis of all meteorological parameters of interest for OSM WQP stations. Here, ‘-’ indicates measurements were not available.
(DOCX)

S2 Table Regression equations in relation to similarity analysis of AT and RH at different heights for WBEA MT stations. Here, ‘-’ indicates measurements were not available.
(DOCX)

S3 Table Regression equations in relation to similarity analysis of SR and PR for WBEA MT stations; and AT, RH, SR, and BP for WBEA ES stations. Here ‘-’ indicates measurements were not available.
(DOCX)
