# Supplementary material for: Quantifying relations and similarities of the meteorological parameters among the weather stations in the Alberta Oil Sands region
Source: PLoS One. 2022 Jan 13;17(1):e0261610. doi: 10.1371/journal.pone.0261610 (PMC8758077; doi:10.1371/journal.pone.0261610)
Supplement: S1 Table — Here, ‘-’ indicates measurements were not available. (DOCX) [file pone.0261610.s001.docx]

**S1 Table.** **Regression equations in relation to similarity analysis of all meteorological parameters of interest for OSM WQP stations. Here, ‘-’ indicates measurements were not available.**

| **Station**  **Pair** | | **AT** | **RH** | **SR** | **BP** | **PR** | **SD** |
| --- | --- | --- | --- | --- | --- | --- | --- |
| C1  vs | C2 | 0.97x-0.33 | 0.90x+7.94 | 1.02x+19.02 | - | 0.57x+0.50 | 0.90x+0.67 |
|  | C3 | 0.99x+0.15 | 0.93x+4.00 | 1.01x+10.68 | - | 0.91x+0.12 | 0.92x-0.21 |
|  | C4 | 0.99x-0.97 | 0.78x+17.99 | 0.99x+10.25 | - | 0.79x+0.40 | 0.92x+0.66 |
|  | C5 | 0.91x+0.23 | 0.79x+15.39 | 0.94x+17.37 | - | 0.82x+0.62 | 0.81x+0.29 |
|  | L1 | 1.01x-0.53 | 0.78x+16.61 | - | - | 0.62x+0.26 | - |
|  | L2 | 1.00x+0.03 | 0.92x+7.19 | - | - | 0.84x+0.18 | - |
| C2  vs | C3 | 1.02x+0.52 | 0.87x+6.70 | 0.91x-1.36 | 0.75x+24.90 | 0.63x+0.34 | 0.96x+0.82 |
|  | C4 | 1.02x-0.58 | 0.82x+13.30 | 0.90x-4.98 | 0.71x+29.75 | 0.83x+0.32 | 1.01x+1.44 |
|  | C5 | 0.93x+0.59 | 0.82x+11.74 | 0.84x+4.80 | 0.62x+35.04 | 0.65x+0.77 | 0.86x+1.21 |
|  | L1 | 1.04x-0.15 | 0.76x+18.20 | - | - | 0.51x+0.29 | - |
|  | L2 | 1.02x+0.31 | 0.88x+9.21 | - | - | 0.56x+0.40 | - |
| C3  vs | C4 | 1.00x-1.11 | 0.80x+17.02 | 0.96x+1.83 | 0.84x+15.88 | 0.66x+0.49 | 0.89x+2.14 |
|  | C5 | 0.92x+0.10 | 0.86x+11.20 | 0.92x+7.85 | 0.74x+22.37 | 0.77x+0.63 | 0.84x+1.02 |
|  | L1 | 1.02x-0.71 | 0.78x+17.35 | - | - | 0.57x+0.27 | - |
|  | L2 | 1.00x-0.29 | 0.94x+6.44 | - | - | 0.62x+0.32 | - |
| C4  vs | C5 | 0.91x+1.12 | 0.84x+10.47 | 0.91x+11.69 | 0.87x+9.97 | 0.58x+0.75 | 0.84x+0.25 |
|  | L1 | 1.02x+0.42 | 0.88x+8.26 | - | - | 0.64x+0.13 | - |
|  | L2 | 1.00x+0.82 | 0.96x+1.99 | - | - | 0.56x+0.38 | - |
| C5  vs | L1 | 1.09x-0.82 | 0.67x+24.35 | - | - | 0.38x+0.31 | - |
|  | L2 | 1.08x-0.40 | 0.83x+12.75 | - | - | 0.4x+0.44 | - |
| L1 vs | L2 | 0.99x+0.51 | 1.03x-2.07 | - | - | 0.78x+0.24 | - |
