# Supplementary material for: Quantifying relations and similarities of the meteorological parameters among the weather stations in the Alberta Oil Sands region
Source: PLoS One. 2022 Jan 13;17(1):e0261610. doi: 10.1371/journal.pone.0261610 (PMC8758077; doi:10.1371/journal.pone.0261610)
Supplement: S2 Table — Here, ‘-’ indicates measurements were not available. (DOCX) [file pone.0261610.s002.docx]

**S2 Table.** **Regression equations in relation to similarity analysis of AT and RH at different heights for WBEA MT stations. Here, ‘-’ indicates measurements were not available.**

| **Station Pair** | | **AT** | | | | **RH** | | | |
| --- | --- | --- | --- | --- | --- | --- | --- | --- | --- |
|  |  | **2m** | **16m** | **21m** | **29m** | **2m** | **16m** | **21m** | **29m** |
| JP104  vs | JP107 | 1.03x-0.78 | 1.02x-0.31 | 1.02x-0.50 | 1.03x-0.44 | 0.85x+10.10 | 0.86x+7.78 | 0.87x+7.70 | 0.79x+12.58 |
|  | JP201 | 0.87x-2.16 | 0.93x-1.09 | 0.92x-0.95 | 0.92x-0.55 | 0.87x+11.88 | 0.86x+12.58 | 0.87x+12.72 | 0.84x+13.68 |
|  | JP213 | 1.00x-1.63 | 1.00x-1.61 | 0.99x-1.45 | 1.00x-1.59 | 0.88x+10.10 | 0.89x+9.30 | 0.89x+9.79 | 0.82x+13.54 |
|  | JP311 | 0.95x+0.14 | 0.95x+0.28 | 0.95x+0.22 | 0.95x+0.18 | 0.88x+9.05 | 0.90x+6.94 | 0.91x+6.67 | 0.84x+11.55 |
|  | JP316 | 0.95x-0.60 | 0.96x-0.23 | 0.96x-0.29 | 0.96x-0.24 | 0.83x+14.05 | 0.85x+11.48 | 0.87x+11.07 | 0.79x+15.56 |
| JP107  vs | JP201 | 0.81x-1.71 | 0.89x-0.78 | 0.88x-0.46 | 0.88x-0.12 | 0.81x+15.94 | 0.77x+19.93 | 0.79x+19.12 | 0.81x+17.26 |
|  | JP213 | 0.96x-1.02 | 0.96x-1.39 | 0.96x-1.06 | 0.96x-1.18 | 0.87x+10.89 | 0.88x+11.84 | 0.88x+11.34 | 0.88x+10.98 |
|  | JP311 | 0.90x+0.83 | 0.91x+0.54 | 0.91x+0.68 | 0.91x+0.58 | 0.82x+13.66 | 0.83x+13.34 | 0.83x+12.83 | 0.83x+13.40 |
|  | JP316 | 0.89x+0.02 | 0.92x+0.05 | 0.92x+0.19 | 0.92x+0.15 | 0.79x+17.04 | 0.81x+16.00 | 0.82x+15.37 | 0.82x+15.65 |
| JP201  vs | JP213 | 0.64x+0.27 | 1.03x-0.41 | 1.04x-0.36 | 1.06x-0.96 | 0.69x+21.92 | 0.76x+16.07 | 0.77x+14.56 | 0.76x+15.16 |
|  | JP311 | 0.67x+2.21 | 0.99x+1.40 | 1.00x+1.24 | 1.02x+0.77 | 0.74x+17.07 | 0.83x+8.97 | 0.82x+9.42 | 0.85x+8.66 |
|  | JP316 | 0.63x+1.53 | 1.00x+0.91 | 1.01x+0.79 | 1.02x+0.36 | 0.66x+24.44 | 0.75x+16.02 | 0.74x+16.13 | 0.77x+15.04 |
| JP213  vs | JP311 | 0.92x+1.75 | 0.94x+1.87 | 0.95x+1.66 | 0.94x+1.76 | 0.83x+11.08 | 0.83x+10.06 | 0.83x+10.49 | 0.83x+10.80 |
|  | JP316 | 0.93x+0.94 | 0.96x+1.34 | 0.96x+1.09 | 0.96x+1.31 | 0.87x+10.38 | 0.88x+7.84 | 0.88x+8.62 | 0.88x+8.82 |
| JP311 vs | JP316 | 0.99x-0.72 | 1.00x-0.49 | 1.00x-0.50 | 1.00x-0.37 | 0.85x+11.68 | 0.87x+10.29 | 0.87x+10.33 | 0.87x+10.15 |
