# Supplementary material for: Quantifying relations and similarities of the meteorological parameters among the weather stations in the Alberta Oil Sands region
Source: PLoS One. 2022 Jan 13;17(1):e0261610. doi: 10.1371/journal.pone.0261610 (PMC8758077; doi:10.1371/journal.pone.0261610)
Supplement: S3 Table — Here ‘-’ indicates measurements were not available. (DOCX) [file pone.0261610.s003.docx]

**S3 Table.** **Regression equations in relation to similarity analysis of SR and PR for WBEA MT stations; and AT, RH, SR, and BP for WBEA ES stations. Here ‘-’ indicates measurements were not available.**

| **WBEA MT stations** | | | | **WBEA ES stations** | | | | | |
| --- | --- | --- | --- | --- | --- | --- | --- | --- | --- |
| **Station Pair** | | **AT** | **RH** | **Station Pair** | | **AT** | **RH** | **SR** | **BP** |
| JP104  vs | JP107 | 0.90x+14.78 | - | JE306  vs | JE308 | 0.93x-0.63 | 0.85x+12.96 | 0.93x+31.87 | 0.46x+50.33 |
|  | JP201 | 1.11x+192.74 | - |  | JE312 | 0.96x-0.45 | 0.97x+3.82 | 0.90x+16.60 | 0.36x+60.80 |
|  | JP213 | 0.90x+17.54 | - |  | JE316 | 0.94x-0.24 | 0.85x+12.60 | 0.87x+36.6 | 0.45x+51.82 |
|  | JP311 | 0.94x+16.10 | - |  | JE323 | 0.96x-0.94 | 0.91x+9.26 | 0.77x+15.77 | 0.45x+51.91 |
|  | JP316 | 0.90x+18.43 | - |  | R2 | 0.96x+0.43 | 0.94x+4.53 | 0.86x+15.5 | 0.52x+46.13 |
| JP107  vs | JP201 | 0.90x+19.77 | - | JE308  vs | JE312 | 1.00x+0.23 | 0.91x+5.75 | 0.8x+22.42 | 0.23x+73.84 |
|  | JP213 | 0.92x+10.51 | 0.30x+0.44 |  | JE316 | 0.98x+0.44 | 0.82x+12.30 | 0.79x+39.46 | 0.31x+65.76 |
|  | JP311 | 0.95x+13.02 | 0.28x+0.45 |  | JE323 | 1.00x-0.28 | 0.89x+7.76 | 0.7x+15.24 | 0.32x+65.96 |
|  | JP316 | 0.91x+13.08 | 0.19x+0.54 |  | R2 | 0.99x+1.05 | 0.89x+5.71 | 0.8x+11.94 | 0.27x+71.35 |
| JP201  vs | JP213 | 0.82x+22.77 | - | JE312  vs | JE316 | 0.97x+0.20 | 0.86x+10.71 | 0.92x+27.73 | 0.61x+36.79 |
|  | JP311 | 0.88x+18.55 | - |  | JE323 | 0.99x-0.48 | 0.89x+8.47 | 0.80x+10.96 | 0.62x+36.86 |
|  | JP316 | 0.83x+22.42 | - |  | R2 | 0.98x+1.03 | 0.66x+22.19 | 0.88x+13.84 | 1.17x-15.44 |
| JP213  vs | JP311 | 0.92x+15.53 | 0.28x+0.48 | JP316  vs | JE323 | 1.01x-0.72 | 0.93x+5.11 | 0.73x+15.68 | 0.36x+61.96 |
|  | JP316 | 0.94x+10.30 | 0.28x+0.47 |  | R2 | 0.99x+0.68 | 0.91x+4.20 | 0.84x+9.63 | 0.36x+63.02 |
| JP311 vs | JP316 | 0.90x+11.25 | 0.22x+0.45 | JP323 vs | R2 | 0.97x+1.52 | 0.90x+5.08 | 0.83x+48.57 | 0.74x+26.14 |
